# Ninety day mortality and its predictors after primary shoulder arthroplasty: an analysis of 4,019 patients from 1976-2008

**DOI:** 10.1186/1471-2474-12-231

**Published:** 2011-10-12

**Authors:** Jasvinder A Singh, John W Sperling, Robert H Cofield

**Affiliations:** 1 Medicine Service and Center for Surgical Medical Acute care Research and Transitions (C-SMART), Birmingham VA Medical Center, Birmingham, AL, USA; 2 Department of Medicine, University of Alabama, and Division of Epidemiology, School of Public Health, University of Alabama, Birmingham, AL, USA; 3 Department of Orthopedic Surgery, Mayo Clinic School of Medicine, Rochester, MN, USA

**Keywords:** mortality, shoulder arthroplasty, humeral head replacement, shoulder hemiarthroplasty

## Abstract

**Background:**

Examine 90-day postoperative mortality and its predictors following shoulder arthroplasty

**Methods:**

We identified vital status of all adults who underwent primary shoulder arthroplasty (Total shoulder arthroplasty (TSA) or humeral head replacement (HHR)) at the Mayo Clinic from 1976-2008, using the prospectively collected information from Total Joint Registry. We used univariate logistic regression models to assess the association of gender, age, body mass index, American Society of Anesthesiologist (ASA) class, Deyo-Charlson comorbidity index, an underlying diagnosis and implant fixation with odds of 90-day mortality after TSA or HHR. Multivariable models additionally adjusted for the type of surgery (TSA versus HHR). Adjusted Odds ratio (OR) with 95% confidence interval (CI) were calculated.

**Results:**

Twenty-eight of the 3, 480 patient operated died within 90-days of shoulder arthroplasty (0.8%). In multivariable-adjusted analyses, the following factors were associated with significantly higher odds of 90-day mortality: higher Deyo-Charlson index (OR, 1.54; 95% CI:1.39, 1.70; p < 0.001); a diagnosis of tumor (OR, 16.2; 95%CI:7.1, 36.7); and ASA class III (OR, 3.57; 95% CI:1.29, 9.91; p = 0.01) or class IV (OR, 13.4; 95% CI:2.44, 73.86; p = 0.003). BMI ≥ 30 was associated with lower risk of 90-day mortality (OR, 0.25; 95% CI:0.08, 0.78). In univariate analyses, patients undergoing TSA had significantly lower 90-day mortality of 0.4% (8/2, 580) compared to 1% in HHR (20/1, 411) (odds ratio, 0.22 (95% CI: 0.10, 0.50); p = 0.0003).

**Conclusions:**

90-day mortality following shoulder arthroplasty was low. An underlying diagnosis of tumor, higher comorbidity and higher ASA class were risk factors for higher 90-day mortality, while higher BMI was protective. Pre-operative comorbidity management may impact 90-day mortality following shoulder arthroplasty. A higher unadjusted mortality in patients undergoing TSA versus HHR may indicate the underlying differences in patients undergoing these procedures.

## Background

Shoulder arthroplasty is an effective surgical procedure for the treatment of refractory shoulder pain due to end stage shoulder disease due to osteoarthritis and other causes. It is associated with improvement in shoulder pain, function and quality of life. Total shoulder arthroplasty (TSA) and humeral head replacement (HHR; shoulder hemi-arthroplasty) are the two most common shoulder arthroplasty procedures [1]. The utilization of shoulder arthroplasty is projected to increase by 192% by 2015 [2].

Like any other surgical procedure, there is a small risk of perioperative mortality after shoulder arthroplasty. TSA and HHR are mostly elective procedures, which makes it critical to study and understand the mortality risk with these procedures. Patients and surgeons need this information to assess risk/benefit ratio and make an informed decision and have realistic expectations from shoulder arthroplasty. While estimates of mortality are available, little is known regarding patient risk factors for mortality after shoulder arthroplasty. In our recent study that included patients up to year 2000, the 90-day mortality after shoulder arthroplasty was 0.58% (17 of 2, 953) [3]. Smaller studies, which were likely underpowered, and had shorter follow-up have reported lower mortality: 30-day mortality of 0.4% in 793 TSA patient from Veterans Affairs medical centers [4] and no in-hospital deaths in 994 shoulder arthroplasties in a discharge database from Maryland [5]. In a study of system factors, lower surgeon volume was associated with higher mortality following shoulder arthroplasty [6]; however no patient-level factors have been studied. Mortality within 90-days of arthroplasty could be potentially linked to surgery and/or its complications and is of more interest to the surgeon and the patient, as opposed to long-term mortality (1- or 5-year post-surgery), which may indicate baseline mortality risk, rather than related to the surgery. In this study, our aims were to: (1) determine the risk factors and rate of 90-day mortality following primary shoulder arthroplasty (TSA or HHR); and (2) if data allowed, to assess whether mortality differed by the type of procedure (TSA versus HHR).

## Methods

### Data Source

Every patient undergoing shoulder arthroplasty at the Mayo clinic is followed prospectively by the clinic's Total Joint Registry (originally for hip, but captures all arthroplasties), which has captured every shoulder arthroplasty since 1976 for occurrence of any complication including death [7]. The Total Joint Registry has full-time trained registry staff that follow patient complications using assessments from scheduled clinic follow-up visits and in those failing to return for clinic visits, using mailed questionnaires and telephone calls. Mortality data (vital status and date of death) are obtained from the medical records at the Mayo clinic and other medical centers, Social Security Death Index and/or verbal report of patient's death by patient's spouse/significant other. Numerous high quality outcomes studies have been published using Mayo Clinic Total Joint Registry, including mortality studies in patients with knee and hip arthroplasty [8,9]. The study was approved by the Mayo Clinic Institutional Review Board.

### Study Population and Outcome

All adults who underwent either primary TSA or HHR between January 1976 and December 2008 at the Mayo clinic, Rochester, MN were included. There were no age or diagnosis restrictions. The outcome of interest was all-cause mortality within 90 days of primary TSA or HHR.

### Predictors of Interest

Our main aim was to assess the predictors of 90-day mortality after primary shoulder arthroplasty. Anticipating a few deaths even with a 33-year data, we planned a priori to perform combined analyses for TSA and HHR, and adjust analyses for the type of surgery (TSA versus HHR), since the indications of the two procedures differ. We anticipated that the number of deaths would be too small to analyze the TSA and HHR separately. The predictors of interest included the following:

(1) Demographics: gender; age, < 60, ≥60-70, > 70 [10];

(2) Clinical:

(a) Body Mass Index (BMI) as categorized < 25, 25-29.9 and > 30 Kg/m^2^;

(b) American Society of Anesthesiologist (ASA) scale, categorized as I/II versus III/IV;

(c) Deyo-Charlson Index, a validated measure of medical comorbidity [11], is a summative weighted scale of 17 comorbidities (including cardiac, pulmonary, renal, hepatic disease, diabetes, cancer, HIV etc.), for example, conditions such as myocardial infarction, dementia, peptic ulcer disease etc. each get a score of "1"; diabetes with end-organ damage, hemiplegia, renal disease, malignancy each get a score of "2"; moderate-severe liver disease a score of "3"; metastatic solid tumor and AIDS/HIV each get score of "6" - this was categorized as 0 or ≥1, since the median for this population was zero;

(d) Diagnosis: categorized as rheumatoid arthritis, osteoarthritis, rotator cuff disease, trauma, other; and

(3) Implant fixation (cemented versus not).

### Statistical Analysis

Summary statistics were calculated for continuous and categorical variables as means or proportions. For the main aim assessing predictors of mortality, we assessed univariate associations of predictors of interest (age, gender, BMI, ASA, Deyo-Charlson, diagnosis and implant fixation) with 90-day mortality using logistic regression. For each predictor, we also performed multivariable-adjusted analyses by adjusting analyses for the type of arthroplasty (TSA versus HHR) based on our clinical prediction that arthroplasty type might be associated with mortality. The objective of adjusting for the type of surgery was to assess if each variable of interest (age, gender, Deyo-Charlson comorbidity index, diagnosis and implant fixation) was independently associated with the outcome, after adjusting for the effect of the type of surgery. Since our main objective was to assess mortality risk factors in patients undergoing shoulder arthroplasty, the HHR and TSA groups were combined for analyses. We decided not to perform additional analyses adjusted for multiple variables to avoid over-adjusted models, since the total number of outcomes was < 30. For analysis of the second aim of whether type of surgery had an impact on mortality, univariate associations of type of surgery with mortality were assessed. We also examined each of the multivariable analyses performed for main aim for significance of type of surgery variable, since all models were simultaneously adjusted for this variable. Odds ratios and 95% confidence intervals are presented. A p-value < 0.05 was considered significant.

## Results

### Clinical and Demographic Characteristics

3, 490 patients who underwent 4, 019 shoulder arthroplasties during 1976-2008 were included in this study. Of these, 1, 431 (36%) underwent shoulder hemi-arthroplasty and 2, 588 (64%) underwent TSA. The mean age was 64 years and 56% were women (Table 1).

**Table 1:** Clinical and socio-demographic characteristics of Study Cohort

	Mean (standard deviation) or % (n)
	**3, 480 patients with 4, 019 shoulder arthroplasties (TSA or HHR)**

**Age in years**	65 (13) [median 67, range 18 to 67]
**Female/male**	2, 250 (56%)/1769 (44%)
**Deyo-Charlson index**	1.0 (1.8)
**Body Mass Index (BMI)**^**a**^	29 (6)
**American Society of Anesthesiologist (ASA) **^**b**^	
ASA Class I/II	1, 725 (57%)
ASA Class III	1, 281 (43%)
ASA Class IV	15 (1%)
**Implant fixation**	
Cemented	3, 342 (83%)
Uncemented	677 (17%)
**Underlying Diagnosis**^c^	
Rheumatoid arthritis	685 (17%)
Trauma	867 (22%)
Tumor^d^	182 (5%)
Osteoarthritis	1, 988 (49%)
Rotator cuff disease	177 (4%)
Other^e^	120 (3%)
	

^a ^BMI was available from 9/1/1987 to present on 3, 127 shoulders;

^b ^ASA was available from 11/1/1988 to 2008 on 2, 317 shoulders

^c ^Except for trauma, all other underlying diagnoses are associated with elective arthroplasty

^d^Of the 182 with underlying diagnosis of tumor, 135 (74%) had primary cancer and 47 (26%) had metastatic cancer.

^e ^Other category for underlying diagnosis includes: avascular necrosis, ankylosing spondylitis, psoriatic arthritis, gout, Charcot arthropathy, dislocation, old injury, prior history of septic arthritis

All numbers rounded to nearest whole number and therefore percents may not add to 100%

TSA, Total Shoulder Arthroplasty; HHR, Humeral Head Replacement

### 90-day Mortality

28 of the 3, 480 patient operated died within 90-days of shoulder arthroplasty (0.8%), 20 after HHR and 8 after TSA. The 90-day mortality risk seemed similar across time-periods: 0.4% (1/261) died during 1976-1980; 0.9% during 1981-1990 (9/1, 016); 0.6% during 1991-2000 (7/1, 219); and 0.7% during 2001-2008 (11/1, 495). Due to the small number of events, we were unable to test for the significance of time-trends in 90-day mortality.

Of the 20 HHR patients, 5 died between day 16 and day 30, 6 died between day 31 and day 60, 9 died between day 61 and day 90. Of the 8 TSA patients, 3 died between day 2 and day 30, 4 died between day 31 and day 60 and one died between day 61 and day 90. No patients died within the first 24 hours after HHR or TSA.

### Predictors of 90-day Mortality

In univariate analyses, lower BMI and higher Deyo-Charlson index were each associated with higher odds of 90-day mortality after shoulder arthroplasty (Table 2). ASA class 3 and class 4 were also associated with significantly increased risk of 90-day mortality after shoulder arthroplasty. An underlying diagnosis of tumor was associated with significantly increased 90-day mortality compared to all other diagnoses combined. We also compared elective versus non-elective (trauma) cases. There were no significant differences in risk of mortality between trauma versus other diagnoses (elective).

**Table 2:** Association of clinical variables with 90-day mortality in univariate analyses and multivariable analyses adjusted for each variable of interest and the type of surgery (TSA versus hemiarthroplasty)

		Univariate models		Multivariable models adjusted for surgery type	
	**90 day mortality** **n/N**^**d **^**(%)**	**Odds ratio (95% Confidence interval)**	**univariate** **p-value**	**Odds ratio (95% Confidence interval)**	**p-value**

**Gender**					
Female (n = 2, 250)	14/2, 236 (1%)	1.0 (ref)		1.0 (ref)	
Male (n = 1, 769)	14/1, 755 (1%)	1.27 (0.61, 2.68)	0.52	1.50 (0.71, 3.17)	0.29
					
**Age**					
≤ 60 (n = 1, 255)	6/1, 249 (0.5%)	1.0 (ref)		1.0 (ref)	
61-70 (n = 1, 213)	6/1, 207 (0.5%)	1.04 (0.33, 3.22)	0.95	1.34 (0.43, 4.19)	0.62
≥ 70 (n = 1, 551)	16/1, 535 (1%)	2.17 (0.85, 5.56)	0.11	2.40 (0.94, 6.17)	0.07
					
**Body Mass Index (BMI)**^**a**^					
< 24.9 (n = 809)	13/796 (2%)	1.0 (ref)		1.0 (ref)	
25-29.9 (n = 1, 094)	7/1, 087 (1%)	0.40 (0.16, 1.00)	0.05	0.46 (0.18, 1.16)	0.10
≥ 30 (n = 1, 224)	4/1, 220 (0.3%)	0.20 (0.07, 0.62)	**0.005**	0.25 (0.08, 0.78)	**0.02**
					
**ASA class**^**b**^					
ASA 1, 2 (n = 1, 725)	5/1, 720 (0.3%)	1.0 (ref)		1.0 (ref)	
ASA 3 (n = 1, 248)	15/1, 233 (1%)	4.19 (1.52, 11.55)	**0.006**	3.57 (1.29, 9.91)	**0.01**
ASA 4 (n = 33)	2/31 (6%)	22.19 (4.15, 118.83)	**0.0003**	13.44 (2.44, 73.86)	**0.003**
					
**Deyo-Charlson Index**					
Index = 0 (n = 2, 275)	3/2, 272 (0.1%)	1.0 (ref)		1.0 (ref)	
Index ≥1 (n = 1, 744)	25/1, 719 (1%)	11.01 (3.32, 36.50)	**< 0.001**	9.99 (3.01, 33.19)	**0.0002**
					
**Diagnosis**					
RA, osteoarthritis, trauma and other^c ^(n = 3, 837)	14/3823 (0.4%)	1.0 (ref)		1.0 (ref)	
Tumor (n = 182)	14/168 (8%)	22.76 (10.68, 48.50)	**< 0.001**	16.18 (7.10, 36.85)	**< 0.001**
					
**Diagnosis**					
RA, osteoarthritis, tumor and other^c ^(n = 3152)	23/3152 (1%)	1.0 (ref)		1.0 (ref)	
Trauma (n = 867)	5/867 (1%)	0.79 (0.30, 2.08)	0.63	0.52 (0.20, 1.40)	0.20
					
**Cement**					
No cement (n = 677)	4/673 (1%)	1.0 (ref)		1.0 (ref)	
Cement (n = 3342)	24/3, 318 (1%)	1.22 (0.42, 3.52)	0.72	2.81 (0.94, 8.39)	0.06

Significant p-value in multivariable model are **in bold**.

^a ^BMI was available from 9/1/1987 to present on 3, 127 shoulders;

^b ^ASA was available from 11/1/1988 to 2008 on 2, 317 shoulders

^c ^Other includes the following diagnoses: tumor (n = 182); rest (n = 297) included avascular necrosis, ankylosing spondylitis, psoriatic arthritis, gout, Charcot arthropathy, dislocation, old injury, prior history of septic arthritis

^d ^Discrepancy in numbers was due to loss to follow-up of a few patients

In multivariable models that adjusted for the type of arthroplasty (TSA versus hemiarthroplasty), higher Deyo-Charlson index, ASA class 3 or class 4, an underlying diagnosis of tumor were each still significantly associated with higher 90-day mortality; BMI ≥30 was significantly associated with lower 90-day mortality, compared to BMI < 25 kg/m^2 ^(Table 2).

The significance of associations of Deyo-Charlson index or BMI with mortality did not change when these variables were examined as continuous variables. Each unit increase in BMI and Deyo-Charlson index was associated with odds ratio of 0.91 (0.84, 0.99) (p = 0.02) and 1.54 (1.39, 1.70) (p < 0.001) for 90-day mortality, respectively, when simultaneously adjusted for the type of surgery.

### Differences between TSA and HHR

In univariate analyses, patients undergoing TSA had significantly lower 90-day mortality of 0.4% (8/2, 580) compared to 1% in HHR (20/1, 411) (odds ratio, 0.22 (95% CI: 0.10, 0.50); p = 0.0003). In all multivariable models that adjusted for each variable of interest, type of surgery (TSA versus HHR) remained a significant variable associated with 90-day mortality, with two exceptions, namely the models that adjusted for the Deyo-Charlson index and the underlying diagnosis.

## Discussion

In this study, we found that 90-day mortality following primary shoulder arthroplasty was 0.8% using 33-year data from a larger medical center. This is in agreement with previous studies reporting 0.6% 90-day mortality after shoulder arthroplasty [3] and with 0.4% 30-day mortality following TSA in veterans [4].

Several associations noted in this study are novel and deserve further discussion. Our observation of association of higher comorbidity with higher 90-day mortality following shoulder arthroplasty extends the recent observation of association of Deyo-Charlson index with in-hospital mortality following shoulder [12]. Future studies need to examine which comorbidities are associated with this increased risk. It remains to be seen whether intensive preoperative comorbidity management could lead to reduction in 90-day mortality after shoulder arthroplasty, a rare but feared complication of this elective procedure.

Our finding of association of higher BMI with lower mortality following shoulder arthroplasty is novel. To our knowledge, there is only one published study that assessed association of BMI with mortality in arthroplasty cohort [13]. In a sample of total hip arthroplasty patients, there was no association of BMI with a composite end-point of mortality, readmission, re-operation or intensive care unit admissions [13]. However, the end-point was composite, not mortality. On the other hand, several studies have shown that lower BMI was a risk factor for postoperative complications after hernia surgery [14], postoperative pulmonary complications after cardiac surgery [15], major adverse cardiac events after percutaneous coronary intervention [16] and postoperative adverse cardiac adverse events after hip fracture repair [17]. Studies in non-arthroplasty populations have shown that low BMI is a risk for higher in-hospital postoperative mortality [15] and 30-day mortality after coronary artery bypass grafting [18]. The "obesity paradox" with worse outcomes noted in patients with lower BMI has also been reported in patients with heart failure [19]. A systematic review of cohort studies of patients with coronary artery disease found lower than normal BMI was associated with higher total mortality and cardiovascular mortality compared to patients with normal BMI [20]. To our knowledge, our study is the first study to observe this association in patients who underwent shoulder arthroplasty. Underweight patients are likely to have low caloric and functional reserve compared to overweight/obese patents, which might make them more vulnerable to complications and subsequent mortality, especially in a stressful postoperative period after arthroplasty. It is also possible that obese people who survive to old age (patients who commonly undergo shoulder arthroplasty) have a survival advantage as compared to non-obese and lean patients. Adipose tissue is considered a metabolic organ, and the underlying mechanisms for this association need to be investigated further. This emerging body of evidence suggests a protective role for normal and/or higher BMI with regards to post-surgical survival. We believe this needs further study.

It is not unexpected that 90-day mortality was higher in patients with higher/worse ASA class. This intuitive finding has not previously reported in shoulder arthroplasty patients. This mirrors similar findings in patients undergoing knee arthroplasty [21]. A higher mortality was also noted in patients with an underlying diagnosis of cancer, which is intuitive.

We found that TSA was associated with lower mortality compared to HHR. This is at least partially due to differences in indications for TSA versus HHR. The mortality risk difference was significant in all multivariable models except two models that simultaneously adjusted for: (1) comorbidity and surgery type and; (2) underlying diagnosis and surgery type. This implies that higher mortality in primary TSA patients compared to HHR was due to higher comorbidity and differences in the underlying diagnoses between TSA and HHR cohorts. This is an important observation that needs to be accounted for in future studies comparing mortality between TSA and HHR cohorts.

Our study has several limitations. BMI and ASA data were not available for the entire study duration, since these were captured starting in 1987/88. However, for both variables, significant associations were detected implying that we had adequate power to examine strong associations. We included a large sample size, but found only 28 events during the 33-year study period, which may have led to type II error, i.e., missing a significant association due to small sample size when such an association really existed. Due to a small sample size, the multivariable models were only adjusted for the surgery type; additional adjustment would have led to over-adjusted models. Residual confounding is possible due to cohort study design, despite our attempts to incorporate several important patient-level factors. It is unlikely that any randomized study in arthroplasty will be large enough to study mortality rates, therefore these analyses are likely to be performed in cohort or case-control studies. The cohort characteristics are similar to those reported in other studies, implying that findings from our study may be generalizable to other settings. We attempted review of medical records but were unable to find the cause of death for most patients, related to one or more of the following reasons: (1) Total joint registry captures the date, but not cause of death; (2) most shoulder arthroplasties, and therefore most deaths occurred in patients referred to the medical center, for whom the cause of death was not documented in medical records; (2) autopsy data were missing for most patients.

## Conclusions

In summary, we found that 90-day mortality following primary shoulder arthroplasty was low at 0.80%. Several patient-level factors, such as higher comorbidity, underlying diagnosis of tumor, higher/worse ASA class, lower BMI were risk factors for 90-day mortality after primary shoulder arthroplasty. Future studies need to examine if optimization of preoperative comorbidity management can further reduce the rare fatality after shoulder arthroplasty.

## Competing interests

The authors declare that they have no competing interests. J.A.S. has received speaker honoraria from Abbott; research and travel grants from Takeda, Savient, Wyeth and Amgen; and consultant fees from URL pharmaceuticals, Novartis and Savient. J.S. has received royalties from Aircast and Biomet, consultant fees from Tornier and owns stock in Tornier. R.C. has received royalties from Smith and Nephew.

## Authors' contributions

JAS drafted the initial study protocol, revised protocol, prepared IRB application, reviewed the data abstraction and data analyses, prepared the first draft of the manuscript, and revised the manuscript. JWS provided comments on the protocol, reviewed the data abstraction and data analyses, provided comments on the manuscript and approved the final manuscript. RHC provided comments on the protocol, reviewed the data abstraction and data analyses, provided comments on the manuscript and approved the final manuscript. All authors have read and approved the manuscript.

## Pre-publication history

The pre-publication history for this paper can be accessed here:

http://www.biomedcentral.com/1471-2474/12/231/prepub
